# Molecular Evaluation of Traditional Chicken Farm-Associated Bioaerosols for Methicillin-Resistant *Staphylococcus aureus* Shedding

**DOI:** 10.3390/antibiotics10080917

**Published:** 2021-07-28

**Authors:** Chi-Wei Tao, Jung-Sheng Chen, Bing-Mu Hsu, Suprokash Koner, Tung-Che Hung, Han-Ming Wu, Jagat Rathod

**Affiliations:** 1 Department of Internal Medicine, Cheng Hsin General Hospital, Taipei 112401, Taiwan; chf105015@chgh.org.tw; 2 Department of Environmental Engineering and Health, Yuanpei University of Medical Technology, Hsinchu 611310, Taiwan; 3 Department of Medical Research, E-Da Hospital, Kaohsiung 824005, Taiwan; ed113187@edah.org.tw; 4 Department of Earth and Environmental Sciences, National Chung Cheng University, Chiayi 621301, Taiwan; suprokashkoner@alum.ccu.edu.tw; 5 Department of Biomedical Sciences, National Chung Cheng University, Chiayi 621301, Taiwan; 6 Department of Infectious Diseases, Ditmanson Medical Foundation Chiayi Christian Hospital, Chiayi 600566, Taiwan; 07627@cych.org.tw; 7 Family Medicine Department, Asia University Hospital, Taichung 413505, Taiwan; D52175@auh.org.tw; 8 Department of Earth Sciences, National Cheng Kung University, Tainan 701401, Taiwan; jagat2006@gmail.com

**Keywords:** methicillin-resistant *Staphylococcus aureus* (MRSA), chicken farm, bioaerosol, molecular typing, toxin genes, multidrug resistance (MDR)

## Abstract

The outbreak of airborne pathogens, such as methicillin-resistant *Staphylococcus aureus* (MRSA) through bioaerosol, and their molecular characterization around domestic poultry farming areas, was not completely understood. This imposes risk of a MRSA-associated health threat for the relevant livestock food production units. To address this issue, the present study investigated the role of bioaerosol in transmitting MRSA strains in poultry house settings by combining molecular typing, phylogenetic classification, antibiotic susceptibility, and virulence gene distribution patterns. The present study highlights that all 18 bioaerosol and stool samples collected were MRSA positive, with a unique set of virulence factors. Out of 57 isolated MRSA isolates, 68.4% and 19.3% consisted of *SCCmec* I and IV elements, respectively, which are commonly linked with hospital-acquired and livestock-associated MRSA strains. It is worth noting that the exfoliative toxin *eta* and *etb* genes were carried by 100% and 70.2% of all isolates, respectively. Only 17.5% of strains showed the presence of enterotoxin *entC*. These MRSA isolates were resistant to chloramphenicol (C), ciprofloxacin (CIP), clindamycin (DA), erythromycin (E), and tetracycline (T), signifying their multi-drug resistance traits. A cluster of phylogenetic analysis described that 80.7% and 15.8% of total isolates belonged to *Staphylococcus aureus* protein A (*spa*) type t002 and t548. Whereas 3.5% were reflected as a new *spa* type. Additionally, as per the chi-squared test score value, these two *spa* types (t002 and t548) have a distribution correlation with HA-MRSA and LA-MRSA in all the samples (*p* < 0.005, chi-squared test; degree of freedom = 1). Ultimately, this study highlights the prevalence of MRSA colonization in the conventional poultry farm environment, showing the risk of bioaerosol transmission, which needs epidemiological attention and prevention strategies.

## 1. Introduction

Methicillin-resistant *Staphylococcus aureus* (MRSA) is a Gram-positive pathogen that could proliferate to a virulent level in the air, and the utmost opportunistic ESKAPE (*Enterococcus faecium*, *Staphylococcus aureus*, *Klebsiella pneumoniae*, *Acinetobacter baumannii*, *Pseudomonas aeruginosa*, and *Enterobacter* spp.) group of pathogens reported worldwide, including Asia [1]. These pathogens are linked to nosocomial infection, and present in versatile natural environments and domestic livestock farming niches [2,3]. The severity and prevalence of MRSA are highly correlated due to the acquisition of antimicrobial resistance characteristics against various groups of drugs, such as β-lactam [4]. MRSA can cause disease in a healthy individual via animate and inanimate vectors, such as infected persons and livestock carriers, contaminated soil, water, and air (e.g., bioaerosols), or through other direct contact [5]. Bioaerosol transmission has significantly been reported as the most potential transmission mode for spreading infection where these native pathogens could disseminate at a level virulent enough in the air [6,7,8]. Under this transmissive mode, airborne MRSA could spread and dominate around neighboring live-stock settings to pose a serious threat for personnel working in farms and livestock, or both [8,9,10]. This could escalate the possibility of economic loss. Eventually, the risk of MRSA colonization within a livestock-associated food production system is also an emerging biosafety and safe-production challenge [11]. Therefore, there is an urgent need to prioritize the identification of MRSA from the chicken source, to define this occupational and public health concern [12]. Besides that, due to multidrug efflux pump and enzymatic degradation mechanisms, MRSA colonies have shown diverse multidrug resistance properties (MDR), which is the highest therapeutic obstacle to their infection treatment [13].

Due to the presence of a tiny mobile genetic element called *mecA* in different classes of Staphylococcal cassette chromosome *mec* (*SCCmec* I to XIV) in the whole genome, this translates to the PBP2a protein (penicillin-binding protein 2a), which defines these *Staphylococcal* *aureus* isolates as MRSA strains with methicillin resistance [14,15]. Moreover, widespread virulence factor genes, such as Panton-Valentine leukocidin (PVL) and enterotoxins (*entA*~*E*), toxic shock syndrome toxin-1 (*tsst-1*), and exfoliative toxin (*eta* and *etb*) could escalate their severity of infection [16]. Broadly, these MRSA strains are categorized into three major epidemiolocal categories, (I) HA-MRSA (hospital-associated MRSA), (II) CA-MRSA (community-associated MRSA), (III) LA-MRSA (livestock-associated MRSA) [10,17]. Briefly, HA-MRSA strains typically carry *SCCmec* elements I–III, whereas CA-MRSA *SCCmec* elements are comprised of type IV, V, or VII + PVL genes as genetic signatures [18]. Whether several LA-MRSA strains can convey any of the *SCCmec* elements’ categorical classes (that are allied with CA-MRSA or HA-MRSA) is still an ongoing investigation [19].

Recently, various molecular characterizations that can differentiate between different types of epidemiologically dominant MRSA strains are being widely used. Among these emerging molecular methods, the *S. aureus* protein A (*spa*) encrypting gene typing is a low-cost rapid tool that has the prominent ability to categorize the epidemiology of MRSA [14]. The variable region (X-region) of the *spa* gene is always targeted for its sequence analysis [20]. MRSA isolates’ profiling through the *mecA* gene is another commonly used genotyping method known as *SCCmec* typing. It is specifically used for the detection of nosocomial infections [21]. Furthermore, the virulence factor linked genes identification can provide some explanation on the strain of infection by *S. aureus.* Cumulatively, all of this information could be vital for the action plan of curbing MRSA spread [22].

Compared to other pathogens, recent reports have suggested a significant transmission of MRSA through the air, which were sampled at a hospital and other health care facilities, animal husbandry units, and domestic waste-water treatment plants [23]. Bioaerosols from traditional and modern farms are known to play a role in the spread of emerging pathogens [24,25]. Although previous reports have shown bioaerosol-based MRSA transmission from the animal husbandry units [26,27,28,29,30,31], there is a lacuna about the characterization of poultry farm bioaerosol-associated MRSA, especially in the case of MRSA-associated risk in traditional farms of Taiwan. Such traditional farms are in close and continuous dynamic interactions with the human population and allow sporadic infection throughout different seasons, and could lead to an outbreak. In this context, this study primarily emphasizes molecular and virulence gene signatures distribution and epidemiology of MRSA strains in the bioaerosol samples around poultry farm area in South-Taiwan. We used *SCCmec* and *spa* typing including MDR pattern, and PCR-based virulence genes identification tools to identify unique molecular distribution as well as prevalence of HA-type in the poultry settings of Taiwan.

## 2. Results

### 2.1. Odorous Compounds and MRSA Prevalence in the Environment

The anemometer data showed that the frequent direction of wind in pre-and post-winter seasons was from the southeast at the sampling sites (Table 1). Whereas in winter, the direction was from the northwest. The wind speed range around the sampling period was 0.4–2.2 m/s. The concentration range of ammonia and methylamine gas in the chicken shed 1 and 2 (indoor environment) was 2–7 ppm and 5–7 ppm, respectively, and outer exposure area had low concentrations (Supplementary Table S1). The concentration of hydrogen sulfide and mercaptan was below the detection limit at both sites. Furthermore, the total airborne bacteria load range in the chicken shed 1 and 2 indoor areas differed from 1.53 × 10^3^ to 2.63 × 10^3^ CFU/m^3^ and 1.63 × 10^3^ to 2.65 × 10^3^ CFU/m^3^, while at the exposure environment, the range was found as 3.04 × 10^2^ to 7.67 × 10^2^ CFU/m^3^. The positive results of MRSA detection revealed that all collected bioaerosol and fecal samples from the chicken farm area were severely contaminated throughout different time point.

### 2.2. Distribution of SCCmec Elements and MRSA Grouping

*SCCmec* typing was performed on 57 MRSA isolates obtained from sampling (Table 2). Among them, 68.4% of isolates possessed *SCCmec* I, designated as the most dominant *SCCmec* element. Out of the total, 19.3% and 12.3% of isolates contained *SCCmec* IV and *SCCmec* VIII. A total of 39 isolates were linked with HA-MRSA, whereas 11 and 7 strains belonged to LA-MRSA and other MRSA groups, respectively. Out of the 16 isolates from shed 1, 10 were HA-MRSA, carrying only *SCCmec* elements I. Only one belonged to LA-MRSA having *SCCmec* IV, and five strains were from other MRSA classes. Chicken shed 2 possessed a total of 12 isolates that were linked with *SCCmec* I containing HA-MRSA strains, and four isolates had *SCCmec* elements IV, considered LA-MRSA. Exposure plaza environment samples led to a total of 11 strains having *SCCmec* I, which were connected with HA-MRSA; four isolates had *SCCmec* IV of LA-MRSA. For stool samples, a total of 10 MRSA isolates were obtained, out of which six isolated were *SCCmec* I-linked HA-MRSA, 2-*SCCmec* IV of LA-MRSA, and 2-*SCCmec* VII, as other MRSA strains.

### 2.3. Toxin Genes Profiling of MRSA Isolates

The toxic gene test results of 57 isolated MRSA strains are shown in Table 3. All of the isolated strains possess the exfoliative toxin *eta*, of which 70.2% (40/57) possessed *etb* gene, and 17.5% MRSA strains had enterotoxin C (*entC*). None of the MRSA isolates from stool samples carried the enterotoxin C toxin gene. For the exfoliative toxin gene b (*etb*) distribution, the highest detection value was found in the chicken shed 1 indoor air samples (87.5%), as well as 70% of stool sample isolates, and 66.7% isolates from outdoor air samples.

### 2.4. Antimicrobial Susceptibility and MRD Pattern of MRSA Isolates

Antimicrobial susceptibility was assessed using the disk diffusion method. A total of eight antimicrobial agents were checked for all of the isolates. The results are shown in Table 4. All isolated strains were resistant to chloramphenicol (C), ciprofloxacin (CIP), clindamycin (DA), erythromycin (E), and tetracycline (T) (100%). Only 33.3% (19/57) and 12.3% (7/57) were resistant to sulfamethoxazole-trimethoprim (S/T) and rifampicin (RA, 12.3%), respectively. As per Magiorakos et al. [32], multidrug-resistant bacteria (MDRB) is defined as being non-susceptible to ≥ 1 agent in ≥ 3 antimicrobial categories; therefore, all 57 MRSA isolates were MDR strains. As shown in Table 5, there are 37 isolates had five antimicrobial drugs resistance pattern denoted as C-CIP-DA-E-T. Moreover, 13 isolates with C-CIP-DA-E-T-S/T and one isolate with a C-CIP-DA-E-RA-T resistance profile. Critically, six strains showed seven antimicrobial drugs resistance denoted as C-CIP-DA-E-RA-T-S/T pattern.

### 2.5. Spa Typing and Phylogenetic Clustering of MRSA Isolates

**Figure 1:**
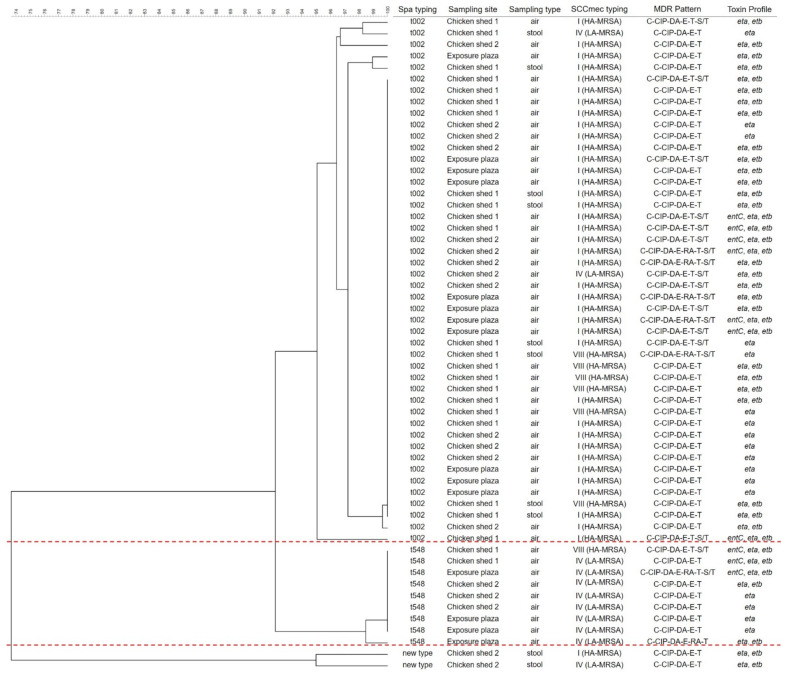
Genetic diversity of MRSA isolates by *Spa* typing combined with MDR pattern, *SCCmec* typing and toxin profile.

*Spa* typing was performed on 57 MRSA isolates; the results are shown in Table 6. Out of 57 MRSA isolates, 46 MRSA strains were nominated as *Spa* type t002 (80.7%), nine (15.8%) isolates belonged to *Spa* type t548 strains, and two strains belonged to *Spa* new type (3.5%). Figure 1 shows a comprehensive phylogenetic analysis of 57 isolated MRSA strains based on *Spa* typing, sampling site and types, *SCCmec* typing, antibiotic resistance, and toxin genes profiles. Furthermore, the phylogenetic classification of all isolates was divided into three main sub-clusters: t002, t548, and new type. The *Spa* t002 type group is typically related to HA-MRSA (*SCCmec* I and VIII), while the *Spa* t548 type group is primarily related to LA-MRSA (*SCCmec* IV). According to the chi-squared test of independence, t002, HA-MRSA vs. t548, LA-MRSA has a distribution correlation (*p* < 0.005, chi-squared test; degree of freedom = 1).

## 3. Discussion

All nine bioaerosol and nine chicken stool samples were collected from three sampling points (chicken 1, 2, and exposure plaza). The concentration of odor-forming gases (such as ammonia and methylamine) were low in outdoor (exposure plaza) air compared to the chicken sheds’ indoor air. Similarly, the total bacteria count in exposure plaza open air was lower than both chicken sheds’ indoor air counts. The previous study detected ammonia (16.8–66.7 mg/m^3^) and methylamine (up to 0.82 mg/m^3^) concentration in the air samples, and 3.2 × 10^9^ CFU/g of total bacteria load in settled dust samples from poultry farm environment [32]. In the present study, the ammonia and methylamine concentrations were in the range of 7–2 ppm and 7–2.5 ppm, respectively. While airborne bacteria were in range of 2.65 × 10^3^–3.04 × 10^2^ CFU/m^3^, which is comparable to other studies from Asia and Europe [33,34]. Ammonia deteriorates the air quality of animals and poultry farms, where it was detected in the range of 0.7–20 ppm [35]. High ammonia concertation could negatively impact livestock production by increasing the severity of disease [36,37,38,39]. Out of the total bacterial load in the farming air, Liu et al. showed that 5.37% of the total 149 *S. aureus* isolates from indoor and outdoor bioaerosol samples were identified to be MRSA [29]. Nasal swab sampling in poultry found that 56.8% of flocks were positive for *S. aureus*, among them 30% were harboring MRSA strains [31]. Likewise, Zhong et al. showed that *S. aureus* can exist in chicken feces and indoor air samples of chicken farm areas at a significant level, and MRSA isolates were also detected in all air and stool samples, which corroborates with our observations [38].

For instance, throughout Europe and North America, the CC398 is a dominant clonal complex of a MRSA strain carrying predominantly *SCCmec* element types IV, V, and occasionally NT [40,41]. Especially in poultry farms, CC9 is regularly detected in the LA-MRSA strain, which is found in pig and poultry houses of Asian countries [40,42]. The sequencing data of strain CC9 indicated that highly versatile types of *SCCmec* elements (III, IV, V, including novel and NT) could exist in their whole genome compared to CC398 [40]. Such genetic diversity of MRSA strains obtained from poultry in Taiwan, which is also uniquely linked to bioaerosol transmission, is studied for the first time in this investigation. *SCCmec* typing results of this study showed a co-occurrence between *SCCmec* type I and IV with HA-MRSA and LA-MRSA. The typical genotype classification of *S. aureus,* such as *SCCmec* I, is linked with HA-MRSA and *SCCmec* IV elements linked to CA-MRSA [43]. However, *SCCmec* type IV and V carrying LA-MRSA lineage are more dominant in the East Asian livestock units [23,40,44]. Recent studies also showed a high prevalence of *SCCmec* IV in the MRSA isolates from poultry and livestock foods, which supports the present study [44,45]. Our previous report about the Chiayi’s river basin and nearby livestock areas revealed that *SCCmec* IV and I were distributed as 64.1% and 15.4%, respectively, wherein LA and HA-MRSA were predominant isolates from water samples, whereas CA and HA-MRSA were predominant isolates from hospital and long-term care facilities environments [46,47]. In the present case, *SCCmec* type I (HA-MRSA) was present in the bioaerosol and stool samples, which could be a future threat to the human population. Likewise, the presence of *SCCmec* IV containing LA-MRSA could colonize livestock animals. Poultry-associated MRSA strain predominantly belonged to CC398 *spa* types, other types of clones were also detected in the diverse geographic region [11]. Here, the *spa* typing and its cluster analysis highlighted that t002 and t548 type strains were linked to *SCCmec* type I and IV elements, respectively. Such distribution of *spa* types was reported from swine farm, indicating t002 and t548 *spa*-type MRSA strains reported from 106 nasal, swabs, and environmental samples [48].

Virulence factors, such as exfoliative toxins (ETs), are epidermolytic in nature. They are serine proteases secreted by *S. aureus* [49]. Around 10% of the MRSA strains possess *eta*; however, a report by Marek et al. showed a limited presence [50,51]. ETs could contribute to the exfoliative epidermitis in pig and Staphylococcal scaled-skin syndrome in humans; however, ETs A, B, and D, originating the human *S. aureus* strain when inoculated in chickens, showed limited or no exfoliative activity [52,53,54]. Our detection results exhibited that 100% of total isolates in this study carried exfoliative toxin *eta* and 70.2% possess *etb*. On contrary, for chicken and duck fecal swab and livestock foods, isolated strains were found negative for exfoliative toxin genes [55,56]. Exfoliative toxin genes were reported in mastitis-infected cow milk samples from Bangladesh [57]. Although a limited risk to poultry, since *eta* and *etb* were linked to human pathogenic MRSA strains, this study highlights a potential occupational and community health challenge [49,58,59]. Therefore, bioaerosol could play a significant role in the transmission of MRSA from livestock farms to adjacent community settings. A total of 17.5% isolates possess *entC* that might increase the risk of Staphylococcal scalded skin or toxic shock syndrome or food poisoning issues [60].

It is noteworthy that all MRSA isolates were MRD. They can typically resist chloramphenicol, ciprofloxacin, clindamycin, erythromycin, and tetracycline. Lu et al. showed that the MRSA isolates from the poultry farm environment can also be multi-drug resistant, which supports present study results [24]. A bioaerosol from livestock farms possess tetracycline and erythromycin resistance *S. aureus* [30]. Taiwan has amended the veterinary drugs control act that restricts the use of multiple antibiotics in food-producing animals. Most of the antibiotics tested in this study are used for human treatment or only for disease control in livestock; therefore, any resistance to these specific antibiotics can be considered a critical epidemiological risk indicator [61]. Strikingly, all 57 MRSA isolates of this study showed multidrug-resistance ability and their virulent profiling suggest that MRSA colonization in the poultry farms have substantial potential to pose a health risk for both human and nearby livestock via bioaerosol transmission.

## 4. Materials and Methods

### 4.1. Study Area and Sampling Information

**Figure 2:**
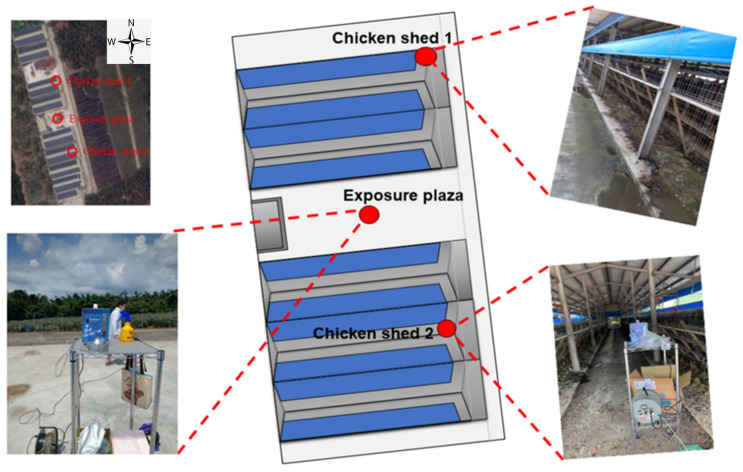
The location map of sampling sites.

The geographical coordinates of the sampling area were 23°35′11.7″ N 120°29′27.3″ E (Figure 2). Here, two chicken sheds, and in between one open exposure plaza, were targeted for environmental bioaerosol collection via a BioStage air sampler. Three bioaerosol and three chicken stool samples were collected at three different times from each targeted sampling point (between June 2019 and March 2020). A total of 18 environmental and fecal samples were collected for further analysis.

### 4.2. Sampling Procedure and Environmental Parameters Analysis

For bioaerosol sampling, the BioStage single-stage cascade impactor (SKC Inc., Blandford Forum Dorset, UK) was placed on a 1.2 m high platform to collect air that could simulate the height of average human breathing. The air sampling flow rate was 28.3 L/min and the sampling time was 10 min. A total 283 L volume of air was used at a time to collect bioaerosol samples. Tryptic soy agar (TSA) with 100 mg/mL cycloheximide was inserted into a BioStage sampler to detect the total number of bacteria count in the environment per volume of air sample (meter cube), and a selective CHROMagar™ MRSA (Paris, France) was also placed in the sampler to screen and isolate MRSA from these environments [62]. Simultaneously, for fecal samples, the stool was collected into sterile specimen bottles. The gas detector tube system (Gastec Inc., Fukayanaka, Japan) was used as per the standard operating procedure to analyze the concentration of odor-producing gases, such as ammonia, methylamine, hydrogen sulfide, and mercaptan (https://www.gastec.co.jp/en/instructionmanual/, accessed on 1 June 2019). Wind speed and direction were also measured using an anemoscope (Puxicoo P6-8232, Shenzhen, China).

### 4.3. Isolation and Selective Cultivation of MRSA Isolates

A total of 1 g of stool sample was taken and added into 9 mL of trypticase soy broth (TSB) with 6.5% NaCl. It was incubated at 37 °C for 16 h. Using a loopful of the TSB enrichment medium, MRSA colonies were isolated on CHROMagar™ MRSA plates (incubated at 37 °C for 24 h). Simultaneously, CHROMagar™ MRSA plates placed in the BioStage sampler system were incubated at 37 °C for 24 h. A single pure mauve color colony from CHROMagar™ MRSA was picked for further analysis by transferring it to Brain-Heart Infusion Broth (BHIB) and incubating at 37 ℃ for 24 h. Subsequently, for confirmation, they were transferred to a moderately selective and differential medium of Baird-Parker Agar and incubated at 37 °C for 24 h. Finally, these pure isolates were grown in BHIB and preserved in 33% glycerol at −20 ℃ for further use. *Staphylococcus aureus* (ATCC25923, ATCC29213) was used as a positive control in this study.

### 4.4. DNA Extraction and PCR Detection of MRSA Isolates

For DNA extraction, first, the freshly grown bacterial cells in the BHIB medium were harvested by centrifugation at 10,000× *g* for 5 min. DNA was extracted by a commercial kit (MagPurix Bacterial DNA Extraction Kit, ZP02006, Taipei, Taiwan) as per their standard protocol. For PCR experiments, the primers and master mix (Fast-Run^TM^ Taq Master Mix with Dye, Springwood, Australia) were mixed with genomic DNA (100–300 μg). The total reaction volume for PCR was 25 μL, and the PCR program conditions of specific detecting genes are described in Supplementary Table S2. *Nuc* and *mecA* genes were utilized to confirmation of MRSA strains. Various molecular elements, such as *mec*, virulence-genes including *SCCmec*, Panton-Valentine leukocidin (PVL), enterotoxins (*entA~E*), toxic shock syndrome toxin-1 (*tsst-1*), and exfoliative toxin (*eta* and *etb*) were confirmed by PCR amplification. The PCR-based *Staphylococcus aureus* Protein A typing (*Spa* typing) data were analyzed by commercial software (BioNumerics, Sint-Martens-Latem, Belgium) for the phylogenetic analysis. the amplicons were confirmed by electrophoresis using 1.5% agarose gel at 110 V for 30 min.

### 4.5. Antibiotic Susceptibility Tests

The disk diffusion method was used for the antibiotic sensitivity test according to the standard protocol by the Clinical and Laboratory Standards Institute (CLSI) [63]. *S. aureus* strains were grown in cation-adjusted Mueller–Hinton broth (CAMHB) at 35 °C. After adjusting to 0.5 McFarland, the medium was evenly spread on Mueller–Hinton agar (MHA) having various antibiotic disks. Plates were incubated at 35 °C for 18–24 h. A total of 8 drugs were checked and their disk concentrations were: chloramphenicol (C, 30 µg), ciprofloxacin (CIP, 5 µg), clindamycin (DA, 2 µg), erythromycin (E, 15 µg), gentamicin (G, 10 µg), rifampicin (RA, 5 µg), sulfamethoxazole-trimethoprim (S/T, 23.75/1.75 µg), and tetracycline (T, 30 µg). Multidrug-resistant bacteria (MDRB) were defined as non-susceptible to ≥ 1 agent in ≥ 3 antimicrobial categories as per Magiorakos et al. [64].

### 4.6. Statistical Analysis

The chi-squared test was performed to prove the statistical significance of the distributional relationship between different isolated MRSA strain spa types with their sampling site, sampling type, *SCCmec* typing, MRD pattern, and existing toxin gene profile groups.

## 5. Conclusions

This pilot study was carried out with different time-specific sampling at two traditional chicken farm sheds and an exposure plaza in South Taiwan, highlighting that all of the bioaerosols collected contained MRSA strains, wherein the total airborne bacterial load was comparatively higher inside the chicken shed environment. Isolated MRSA strains were able to resist multiple antibiotics, such as chloramphenicol, ciprofloxacin (CIP), clindamycin (DA), erythromycin (E), and tetracycline (T), underpinning their MDR characteristics. This study found a high prevalence of exfoliative toxin genes, *eta* and *etb,* in the MRSA isolated strains. The *SCCmec* element profiling showed the predominant occurrence of *SCCmec* I-associated among 68.4% HA-MRSA strains, while only *SCCmec* IV elements were prevalent in 19.3% LA-MRSA isolates. Phytogenic classification by *spa* typing revealed that 46 and 9 MRSA isolates were *Spa* type t002 and t548 strains, respectively. Two isolates were categorized as the new *spa*-type. Additionally, t002 and t548 *spa* types were positively correlated with hospital and livestock allied MRSA infections. Ultimately, multidrug resistant HA-MRSA and LA-MRSA dominance in these samples have enough potential to impose epidemiological risk via bioaerosol transmission through unhygienic poultry practices.

## Figures and Tables

**Table 1:** MRSA prevalence and odorous compounds detection results.

Sampling	Sampling	Wind Speed	Wind	BioStage	MRSA	MRSA
Period	Sites	(m/s)	Direction	(CFU/m^3^)	Air	Stool
1st (June 2019)	Chicken shed 1	N/A	southeast	2.63 × 10^3^	+	+
	Chicken shed 2	N/A	southeast	1.63 × 10^3^	+	+
	Exposure plaza	N/A	southeast	3.04 × 10^2^	+	+
2nd (December 2019)	Chicken shed 1	0.4–0.6	northwest	2.36 × 10^3^	+	+
	Chicken shed 2	0.4–0.6	northwest	2.65 × 10^3^	+	+
	Exposure plaza	0.4–0.6	northwest	6.86 × 10^2^	+	+
3rd (March 2020)	Chicken shed 1	1.9–2.2	northwest	1.53 × 10^3^	+	+
	Chicken shed 2	1.9–2.0	north	1.81 × 10^3^	+	+
	Exposure plaza	1.0–1.2	west	7.67 × 10^2^	+	+

(N/A = not applicable) (m/s = meter/second).

**Table 2:** *SCCmec* PCR typing results of the 57 MRSA isolate.

Sampling Sites	*SCCmec* I	*SCCmec* II	*SCCmec* III	*SCCmec* IV	*SCCmec* V	*SCCmec* VIII	PVL	HA-MRSA (I, II, III)	CA-MRSA (IV + PVL, V + PVL)	LA-MRSA (IV, V)	Others
Chicken shed 1 (*n* = 16)	10 (62.5%)	0 (0%)	0 (0%)	1 (6.3%)	0 (0%)	5 (31.25%)	0 (0%)	10 (62.5%)	0 (0%)	1 (6.3%)	5 (31.25%)
Chicken shed 2 (*n* = 16)	12 (75%)	0 (0%)	0 (0%)	4 (25%)	0 (0%)	0 (0%)	0 (0%)	12 (75%)	0 (0%)	4 (25%)	0 (0%)
Exposure plaza (*n* = 15)	11 (73.3%)	0 (0%)	0 (0%)	4 (26.7%)	0 (0%)	0 (0%)	0 (0%)	11 (73.3%)	0 (0%)	4 (26.7%)	0 (0%)
Stool (*n* = 10)	6 (60%)	0 (0%)	0 (0%)	2 (20%)	0 (0%)	2 (20%)	0 (0%)	6 (60%)	0 (0%)	2 (20%)	2 (20%)
Total MRSA isolates (*n* = 57)	39 (68.4%)	0 (0%)	0 (0%)	11 (19.3%)	0 (0%)	7 (12.3%)	0 (0%)	39 (68.4%)	0 (0%)	11 (19.3%)	7 (12.3%)

**Table 3:** Toxin genes PCR detecting results of the 57 MRSA isolates.

Sampling Sites	*entA*	*entB*	*entC*	*entD*	*entE*	*eta*	*etb*	*tsst-1*
Chicken shed 1 (*n* = 16)	0 (0%)	0 (0%)	5 (31.25%)	0 (0%)	0 (0%)	16 (100%)	14 (87.5%)	0 (0%)
Chicken shed 2 (*n* = 16)	0 (0%)	0 (0%)	2 (12.5%)	0 (0%)	0 (0%)	16 (100%)	9 (56.25%)	0 (0%)
Exposure plaza (*n* = 15)	0 (0%)	0 (0%)	3 (20%)	0 (0%)	0 (0%)	15 (100%)	10 (66.7%)	0 (0%)
Stool (*n* = 10)	0 (0%)	0 (0%)	0 (0%)	0 (0%)	0 (0%)	10 (100%)	7 (70%)	0 (0%)
Total MRSA isolates (*n* = 57)	0 (0%)	0 (0%)	10 (17.5%)	0 (0%)	0 (0%)	57 (100%)	40 (70.2%)	0 (0%)

**Table 4:** Antimicrobial susceptibility results of the 57 MRSA isolates.

Sampling Sites	C	CIP	DA	E	G	RA	S/T	T	MDR
Chicken shed 1 (*n* = 16)	16 (100%)	16 (100%)	16 (100%)	16 (100%)	0 (0%)	0 (0%)	6 (37.5%)	16 (100%)	16 (100%)
Chicken shed 2 (*n* = 16)	16 (100%)	16 (100%)	16 (100%)	16 (100%)	0 (0%)	2 (12.5%)	5 (31.2%)	16 (100%)	16 (100%)
Exposure plaza (*n* = 15)	15 (100%)	15 (100%)	15 (100%)	15 (100%)	0 (0%)	4 (26.7%)	6 (40%)	15 (100%)	15 (100%)
Stool (*n* = 10)	10 (100%)	10 (100%)	10 (100%)	10 (100%)	0 (0%)	1 (10%)	2 (20%)	10 (100%)	10 (100%)
Total MRSA isolates (*n* = 57)	57 (100%)	57 (100%)	57 (100%)	57 (100%)	0 (0%)	7 (12.3%)	19 (33.3%)	57 (100%)	57 (100%)

C: chloramphenicol; CIP: ciprofloxacin; DA: clindamycin; E: erythromycin; G: gentamicin; RA: rifampicin; S/T: sulfamethoxazole-trimethoprim; T: tetracycline; MDR: multidrug resistance.

**Table 5:** MDR pattern profile results of the 57 MRSA isolates.

Sampling Sites	Chicken Shed 1	Chicken Shed 2	Exposure Plaza	Stool	Total MDR Isolates
C-CIP-DA-E-RA-T-S/T (7 antimicrobial drugs)	0	2	3	1	6
C-CIP-DA-E-T-S/T (6 antimicrobial drugs)	6	3	3	1	14
C-CIP-DA-E-RA-T (6 antimicrobial drugs)	0	0	1	0	
C-CIP-DA-E-T (5 antimicrobial drugs)	10	11	8	8	37
Total MDR isolates	16	16	15	10	57

C: chloramphenicol; CIP: ciprofloxacin; DA: clindamycin; E: erythromycin; RA: rifampicin; S/T: sulfamethoxazole-trimethoprim; T: tetracycline; MDR: multidrug resistance.

**Table 6:** *Spa* typing results of the 57 MRSA isolates.

Sampling Sites	t002	t548	New Type
Chicken shed 1 (*n* = 16)	14 (87.5%)	2 (12.5%)	0 (0%)
Chicken shed 2 (*n* = 16)	13 (81.25%)	3 (18.75%)	0 (0%)
Exposure plaza (*n* = 15)	11 (73.3%)	4 (26.7%)	0 (0%)
Stool (*n* = 10)	8 (80%)	0 (0%)	2 (20%)
Total MRSA isolates (*n* = 57)	46 (80.7%)	9 (15.8%)	2 (3.5%)

## Data Availability

The data presented in this study are available upon request from the corresponding author.

## Supplementary Materials

The following are available online at https://www.mdpi.com/article/10.3390/antibiotics10080917/s1, Table S1, Summarization of environmental monitoring background data with bacterial load and MRSA prevalence in collected bioaerosol and stool samples; Table S2, The PCR detecting conditions for strain identification, *Spa* typing, *SCCmec* typing, and enterotoxin characteristics. References [65,66,67,68,69,70,71,72] are cited in the Supplementary Materials.
