# Supplementary material for: Molecular Evaluation of Traditional Chicken Farm-Associated Bioaerosols for Methicillin-Resistant Staphylococcus aureus Shedding
Source: Antibiotics (Basel). 2021 Jul 28;10(8):917. doi: 10.3390/antibiotics10080917 (PMC8388662; doi:10.3390/antibiotics10080917)
Supplement: Supplementary file 1 [file antibiotics-10-00917-s001.zip › antibiotics-1239316-supplementary.pdf]

Table S1. Summarization of environmental monitoring background data with bacterial load and MRSA prevalence in collected bioaerosol and stool samples.

	Samples No (n)	Concentration detection of odor pollutants				Wind direction		MRSA detection rate (%)		Total bacteria count in air
		Ammonia	Methylamine	Hydrogen sulfide	Mercaptan	Summer	Winter	Bioaerosol	Stool	(CFU/m^3)
Chicken shed 1	3	2-7ppm	5-7ppm	N/D	N/D	south-east	north-west	3/3 (100%)	3/3 (100%)	1.53×10^3 to 2.63×10^3
Chicken shed 2	3	2-7ppm	5ppm	N/D	N/D	south-east	north-west, north	3/3 (100%)	3/3 (100%)	1.53×10^3 to 2.65×10^3
Exposure plaza	3	3ppm	2.5ppm	N/D	N/D	south-east	north-west, west	3/3 (100%)	3/3 (100%)	7.67×10^2 to 3.04×10^2

N/D = below detection limit (detection limit for hydrogen sulfide and mercaptan is < 0.05 ppm and < 0.1 ppm)

Table S2. The PCR detecting conditions for strain identification, *Spa* typing, *SCCmec* typing, and enterotoxin characteristics

Target gene	Size	Sequence (5' to 3')	Reaction Materials<br>Final Volume: 25 µl	PCR Condition	Reference
nuc<br>*mecA*	270<br>448	nuc-F 5′-GCGATTGATGGTGATACGGTT-3′<br>nuc-R 5′-AGCCAAGCCTTGACGAACTAAAGC-3′<br>mecA-F 5′-CTCAGGTACTGCTATCCACC-3′<br>mecA-R 5′-CACTTGGTATATCTTCACC-3′	DNA: 100-300 ng<br>Primer: 400 nM<br>nuc FR & mecA FR<br>Master mix: 5 µl	Pre-denaturation: 95°C 5 min<br>Denaturation: 94°C 60s<br>Annealing: 55°C 60s<br>Extension: 72°C 60s<br>D.A.E. Cycles: 30 cycles<br>Final extension: 72°C 10 min	[1,2]
Spa	270	Spa-1113F: 5'- TAAAGACGATCCTTCGGTGAGC -3'<br>Spa-1514R: 5'- CAGCAGTAGTGCCGTTTGCTT -3'	DNA: 100-300 ng<br>Primer: 200 nM<br>Spa FR<br>Master mix: 5 µl	Pre-denaturation: 80°C 5 min<br>Denaturation: 94°C 45s<br>Annealing: 60°C 45s<br>Extension: 72°C 90s<br>D.A.E. Cycles: 35 cycles<br>Final extension: 72°C 10 min	https://spaserver.ridom.de/
SCCmec I<br>SCCmec II<br>SCCmec II, III<br>SCCmec III<br>SCCmec III<br>SCCmec I, II, IV	495<br>284<br>209<br>243<br>414<br>342	CIF2 F2: 5'-TTCGAGTTGCTGATGAAGAAGG-3'<br>CIF2 R2: 5'-ATTTACCACAAGGACTACCAGC-3'<br>KDP F1: 5'-AATCATCTGCCATTGGTGATGC-3'<br>KDP R1: 5'-CGAATGAAGTGAAAGAAAGTGG-3'<br>MECI P2: 5'-ATCAAGACTTGCATTCAGGC-3'<br>MECI P3: 5'-GCGGTTTCAATTCACTTGTC-3'<br>RIF F3: 5'-GTGATTGTTCGAGATATGTGG-3'<br>RIF R9: 5'-CGCTTTATCTGTATCTATCGC-3'<br>RIF F10: 5'-TTCTTAAGTACACGCTGAATCG-3'<br>RIF R13: 5'-GTCACAGTAATTCCATCAATGC-3'<br>DCS F2: 5'-CATCCTATGATAGCTTGGTC-3'<br>DCS R1: 5'-CTAAATCATAGCCATGACCG-3'	DNA: 100-300 ng<br>Primer:<br>400 nM CIF-FR,<br>200 nM KDP-FR,<br>200 nM RIFF3R9<br>400 nM MECI-FR,<br>400 nM RIFF10R13,<br>800 nM DCS-FR<br>Master mix: 5 µl	Pre-denaturation: 94°C 4 min<br>Denaturation: 94°C 30s<br>Annealing: 53°C 30s<br>Extension: 72°C 1 min<br>D.A.E. Cycles: 30 cycles<br>Final extension: 72°C 4 min	[3]
SCCmec V	325	Type V-F: 5'-GAACATTGTTACTTAAATGAGCG-3'<br>Type V-R: 5'-TGAAAGTTGTACCCTTGACACC-3'	DNA: 100-300 ng<br>Primer:<br>100 nM V-FR<br>Master mix: 5 µl	Pre-denaturation: 94°C 5 min<br>Denaturation-1: 94°C 45s<br>Annealing-1: 65°C 45s<br>Extension-1: 72°C 1.5 min<br>D.A.E.-1 Cycles: 10 cycles<br>Denaturation-2: 94°C 45s<br>Annealing-2: 55°C 45s<br>Extension-2: 72°C 1.5 min<br>D.A.E.-2 Cycles: 25 cycles<br>Final extension: 72°C 10 min	[4]
SCCmec VI	134	ccrB4-F: 5'-CGAAGTATAGACACTGGAGCGATA-3'<br>ccrB4-R: 5'-GCGACTCTCTTGGCGTTTA-3'	DNA: 100-300 ng<br>Primer:<br>100 nM FR,<br>Master mix: 5 µl	Pre-denaturation: 95°C 10 min<br>Denaturation: 95°C 30s<br>Annealing: 50°C 30s<br>Extension: 72°C 30s<br>D.A.E. Cycles: 40 cycles<br>Final extension: 72°C 5 min	[5]
SCCmec VII<br>SCCmec VIII	473<br>138	Type VII F: 5'-GTGACGTTGATATTGCAGTGGT-3'<br>Type VII R: 5'-TGAAGAAGTTTGTTCCGCGT-3'<br>Type VIII F: 5'-AGCGACGATGAACAACACCGCTACTTACTCAA-3'<br>Type VIII R: 5'-TTGGTTGAGAATGAGAACAGTGGTAAGATC-3'	DNA: 100-300 ng<br>Primer:<br>400 nM FR<br>Master mix: 5 µl	Pre-denaturation: 95°C 2 min<br>Denaturation: 95°C 30s<br>Annealing: 54°C 1 min<br>Extension: 72°C 1 min 20s<br>D.A.E. Cycles: 35 cycles<br>Final extension: 72°C 7 min	[6]
PVL	433	PVL-1: 5'-ATCATTAGGTAAAATGTCTGGACATGATCCA-3'<br>PVL-2: 5'-GCATCAAGTGTATTGGATAGCAAAAGC-3'	DNA: 100-300 ng<br>Primer:<br>400 nM FR<br>Master mix: 5 µl	Pre-denaturation: 94°C 5 min<br>Denaturation: 94°C 40s<br>Annealing: 53°C 40s<br>Extension: 72°C 1 min<br>D.A.E. Cycles: 35 cycles<br>Final extension: 72°C 10 min	[7]

entA<br>*entB*<br>*entC*<br>*entD*<br>*entE*<br>*tsst-1*<br>*eta*<br>*etb*	121<br>478<br>459<br>384<br>495<br>271<br>464<br>200	entA-F: 5′-TTGGAACGGTTAAAACGAA-3′<br>entA-R: 5′-GAACCTTCCCATCAAAAACA-3′<br>entB-F: 5′-TCGCATCAAACTGACAAACG-3′<br>entB-R: 5′-GCAGGTACTCTATAAGTGCC-3′<br>entC-F: 5′-GGAGGAATAACAAAACATGAAGG-3′<br>entC-R: 5′-AAAGGCAAGCACCGAAGTAC-3′<br>entD-F: 5′-TGGTGGTGAAATAGATAGGAC-3′<br>entD-R: 5′-TGAAGGTGCTCTGTGGATAAT-3′<br>entE-F: 5′-TGGTAGCGAGAAAAGCGAAG-3′<br>entE-R: 5′-TGTAAATAATGCCTTGCCTGAA-3′<br>tsst-1-F: 5′-CTGGTATAGTAGTGGGTCTG-3′<br>tsst-1-R: 5′-AGGTAGTTCTATTGGAGTAGG-3′<br>eta-F: 5′-TTTGCTTTCTTGATTTGGATTC-3′<br>eta-R: 5′-GATGTGTTCGGTTTGATTGAC-3′<br>etb-F: 5′-ACGGCTATATACATTCAATT-3′<br>etb-R: 5′-TCCATCGATAATATACCTAA-3′	DNA: 100-300 ng<br>Primer: 400 nM<br>Primer FR<br>Master mix: 5 µl	Pre-denaturation: 94°C 5 min<br>Denaturation: 94°C 1 min<br>Annealing: 2 min<br>Extension: 72°C 1 min<br>D.A.E. Cycles: 35 cycles<br>Final extension: 72°C 5 min<br>AnnealingTemp.<br>*entA*: 50°C<br>*entB*: 55°C<br>*entC*: 59°C<br>*entD*: 51°C<br>*entE*: 55.5°C<br>*tsst-1*: 54°C<br>*eta*: 54°C<br>*etb*: 50.9°C	[8]

References

1. Brakstad, O.G.; Aasbakk, K.; Maeland, J.A. Detection of *Staphylococcus aureus* by polymerase chain reaction amplification of the *nuc* gene. *Journal of clinical microbiology* **1992**, *30*, 1654-1660.
2. Sakoulas, G.; Gold, H.S.; Venkataraman, L.; DeGirolami, P.C.; Eliopoulos, G.M.; Qian, Q. Methicillin-resistant *Staphylococcus aureus*: comparison of susceptibility testing methods and analysis of mecA-positive susceptible strains. *Journal of clinical microbiology* **2001**, *39*, 3946-3951.
3. Oliveira, D.C.; Lencastre, H.n.d. Multiplex PCR strategy for rapid identification of structural types and variants of the mec element in methicillin-resistant *Staphylococcus aureus*. *Antimicrobial agents and chemotherapy* **2002**, *46*, 2155-2161.
4. Zhang, K.; McClure, J.-A.; Elsayed, S.; Louie, T.; Conly, J.M. Novel multiplex PCR assay for characterization and concomitant subtyping of *staphylococcal cassette chromosome mec* types I to V in methicillin-resistant *Staphylococcus aureus*. *Journal of clinical microbiology* **2005**, *43*, 5026-5033.
5. Chen, L.; Mediavilla, J.R.; Oliveira, D.C.; Willey, B.M.; De Lencastre, H.; Kreiswirth, B.N. Multiplex real-time PCR for rapid *staphylococcal cassette chromosome mec* typing. *Journal of clinical microbiology* **2009**, *47*, 3692-3706.
6. Bhowmik, D.; Das, B.J.; Pandey, P.; Chetri, S.; Chanda, D.D.; Bhattacharjee, A. An array of multiplex PCR assays for detection of s*taphylococcal chromosomal cassette mec* (*SCCmec*) types among staphylococcal isolates. *Journal of microbiological methods* **2019**, *166*, 105733.
7. Asghar, A.H. Molecular characterization of methicillin-resistant *Staphylococcus aureus* isolated from tertiary care hospitals. *Pakistan journal of medical sciences* **2014**, *30*, 698.
8. Fooladi, A.A.I.; Ashrafi, E.; Tazandareh, S.G.; Koosha, R.Z.; Rad, H.S.; Amin, M.; Soori, M.; Larki, R.A.; Choopani, A.; Hosseini, H.M. The distribution of pathogenic and toxigenic genes among MRSA and MSSA clinical isolates. *Microbial pathogenesis* **2015**, *81*, 60-66.
